# Big bodies, blurred lines: the impact of male body size on sensitivity to sexually dimorphic facial features

**DOI:** 10.3389/fpsyg.2025.1531324

**Published:** 2025-04-17

**Authors:** Haoliang Zhu, Shitao Chen, Xue Lei, Zhe Ni, Chengyang Han

**Affiliations:** ^1^Department of Psychology, Wenzhou University, Wenzhou, China; ^2^Department of Psychology and Behavioral Sciences, Zhejiang University, Hangzhou, China; ^3^School of Management, Zhejiang University of Finance and Economics, Hangzhou, China; ^4^Department of Social Psychology, Affiliated Mental Health Center & Hangzhou Seventh People’s Hospital and School of Brain Science and Brain Medicine, Zhejiang University School of Medicine, Hangzhou, China; ^5^Department of Psychology, Jing Hengyi School of Education, Hangzhou Normal University, Hangzhou, China

**Keywords:** body size, sexual dimorphism, gender perception, sensitivity, height

## Abstract

**Introduction:**

Sexual dimorphism plays an important role not only in gender perception but also in social judgment (e.g., dominance). Body size is a key indicator of men’s formidability, influencing their sensitivity to masculinity-based dominance toward other men. However, it remains unclear whether body size also affects men’s sensitivity to sexually dimorphic features in gender perception.

**Methods and results:**

In the current study, we found that men with larger body sizes—operationalized by height, weight, and BMI—exhibited reduced sensitivity to sexually dimorphic facial features during gender judgment. This finding suggests that individual differences (e.g., body size) can modulate men’s sensitivity to the perception of sexually dimorphic traits, potentially cascading into altered sensitivity to social judgments based on these features. In addition, the current study revealed that men showed greater sensitivity to sexually dimorphic features on male faces compared to female faces.

**Discussion:**

Overall, these findings contribute to the literature on individual differences in sensitivity to sexually dimorphic features and their implications for social judgment.

## 1 Introduction

Sexual dimorphism refers to the morphological differences between male and female individuals, playing a critical role in mate selection and social interactions (Jones et al., 2010; Mori et al., 2022). Human sexual dimorphism cues, such as facial masculinity and femininity, reveal vital details about an individual’s reproductive potential, health, and threat potential (Thornhill and Gangestad, 2006; Rhodes et al., 2005; Little et al., 2011, 2015). Accurately identifying and interpreting these cues can significantly impact social judgments, including perceptions of attractiveness and dominance, which essentially influence mate preferences and competitive interactions between people (Han et al., 2022; Richardson et al., 2021).

Sexually dimorphic traits are one of the primary factors that contribute to facial attractiveness in women (Perrett et al., 1998). Understanding the role of sexual dimorphism in women’s facial attractiveness is important for uncovering the complicated mechanisms underlying mate preferences, social judgment, and the evolution of beauty standards (Little et al., 2011). Facial femininity is believed to signal aspects of reproductive health, genetic quality, and fertility in women (Thornhill and Gangestad, 2006; Rhodes et al., 2005). According to evolutionary theories, people are attracted to face features that suggest high genetic fitness because these traits may increase the likelihood of successful reproduction and offspring survival (Buss, 1989; Gangestad and Simpson, 2000). Research on the connection between women’s attractiveness and facial dimorphism has provided strong evidence regarding the impact of these characteristics on mate preferences and social judgment (Han et al., 2020; Little et al., 2011). Both men and women find women with more feminine facial features, such as softer curves, larger eyes, and smaller jaws, tend to be more attractive (Perrett et al., 1998; Jones et al., 2018; Little et al., 2011). These findings indicate that increased femininity in women’s faces enhances their general attractiveness and elicits positive social evaluations.

Sexually dimorphic cues on men’s faces significantly influence their dominant appearance (Richardson et al., 2021; Watkins et al., 2010a,b). Facial masculinity, characterized by prominent jawlines and brow ridges, is associated with perceptions of physical strength and dominance (Windhager et al., 2011; Richardson et al., 2021). These cues are important from an evolutionary perspective as they help people assess possible allies or competitors in social and competitive contexts. Men with more masculine facial features are judged as more dominant and formidable, which can have an impact on social hierarchy, mate preferences, and interpersonal interactions (Oosterhof and Todorov, 2008; Watkins et al., 2010a,b). These insights are supported empirically by studies such as those conducted by Watkins et al. (2010a,b) and Richardson et al. (2021), which show the intricate relationship between men’s facial dimorphism and social perception.

There are individual differences in the social judgment of sexually dimorphic cues. For example, shorter and less dominant men are more sensitive to dominance cues in other men (Watkins et al., 2010a,b). Specifically, it was observed that, during the task of selecting a dominant face from pairs of more or less sexually dimorphic male faces, shorter and less dominant men more frequently selected the face with increased masculine features (Watkins et al., 2010a,b). Moreover, it was found that men’s age and strength also influence their sensitivity to dominance when selecting dominant faces from sexually dimorphic male face pairs (Richardson et al., 2021). In addition, men’s testosterone levels may influence their judgment of the attractiveness of sexually dimorphic cues on women’s faces (Han et al., 2020). Given that men’s height, dominance, strength, age, and testosterone levels are associated with their formidability, it is reasonable to infer that men’s formidability may influence their sensitivity to the social perception of sexually dimorphic cues.

Although previous research has examined individual differences in social judgment (e.g., Watkins et al., 2010a,b; Richardson et al., 2021; Han et al., 2020), these studies have primarily focused on manipulating sexually dimorphic cues rather than investigating the specific traits that correspond to various social judgments (e.g., attractiveness and dominance). This limits our understanding of how individual differences in sensitivity to these cues may impact different aspects of social judgment. It is crucial to discern whether the observed variation in sensitivity to social judgment is primarily driven by sensitivity to sexually dimorphic cues or if the observed variation reflects a broader sensitivity to the psychological processes involved in social judgment. Surprisingly, to the best of the authors’ knowledge, no one has directly studied individual differences in recognizing and accurately perceiving sexually dimorphic cues.

The current study aimed to investigate individual differences in sensitivity to sexually dimorphic cues that signal gender. Men’s formidability may influence their social perception of sexually dimorphic cues (Watkins et al., 2010a,b; Richardson et al., 2021; Han et al., 2020). Body size is one of the important indicators of men’s formidability (Sell et al., 2009). In the current study, we investigated whether men’s body size influences their sensitivity to sexually dimorphic cues in gender perception. Given that taller and more dominant men are less likely to choose masculine male faces as the more dominant ones (Watkins et al., 2010a,b), we predicted that men with larger body sizes would be less sensitive to sexually dimorphic cues in a gender perception task.

Furthermore, we conducted an exploratory analysis to investigate whether men’s sensitivity to sexually dimorphic features would be influenced by facial sex (i.e., the sexual dimorphism of the face); however, this was not the primary goal of the current study. There is no existing literature on this topic, and we formulated our prediction as follows. Since misjudging other men’s sexually dimorphic features would carry greater costs (e.g., potential harm in intrasexual competition) for men than misjudging women’s sexually dimorphic features (e.g., missing a potential mating opportunity in intersexual selection), we predicted that men would be more sensitive to sexually dimorphic cues on men’s faces compared to those on women’s faces.

## 2 Methods

### 2.1 Participants

In total, 112 heterosexual men, aged between 18 and 28 years (*M* = 21.66, *SD* = 2.02), were recruited from the student population at a local university. A power analysis indicated that a sample size of 112 participants would be sufficient to achieve a power of 80% for detecting a small-to-medium effect size (Cohen *f*^2^ = 0.1). Individuals who had any psychiatric disorders and those taking psychotropic medications were not eligible to participate. All the participants had normal or corrected-to-normal vision. The participants received CNY¥ 40 per hour as compensation for their participation. The experimental procedures were approved by the University Ethics Committee and complied with the principles of the Declaration of Helsinki.

### 2.2 Stimuli

#### 2.2.1 Face images collection

Face images of 50 Chinese men (mean age = 24.39 years, SD = 3.52 years) and 50 Chinese women (mean age = 23.94 years, SD = 2.63 years) were collected. The face images were captured under standard lighting conditions, at a constant distance, and with a neutral expression, using a Canon EOS3000D camera. The portrait mode setting was consistently applied to ensure uniformity in image quality and depth of field. The participants were compensated according to the standard rate for behavior studies.

#### 2.2.2 Generating androgynous (gender-neutral) faces

Five male faces and five female faces were randomly selected from the collected image pool (with no repetitions) to synthesize an androgynous face using Psychomorph (Tiddeman et al., 2001), which was used to average the shape, texture, and color information. This process was repeated multiple times, resulting in 50 androgynous faces. Hair and clothing were removed from the images to minimize distractions. Based on a pilot study, we selected five relatively gender-neutral faces as the face stimuli for the current study. Notably, the five androgynous faces were synthesized from 50 individual faces, with no repeated face identities.

#### 2.2.3 Generating prototype (i.e., average) faces

A male prototype face and a female prototype face were generated from the collected faces of the 50 men and 50 women included in the study, respectively, by averaging their shape, texture, and color information using Psychomorph.

#### 2.2.4 Generating face stimuli with standardized sex information

**Figure 1:**
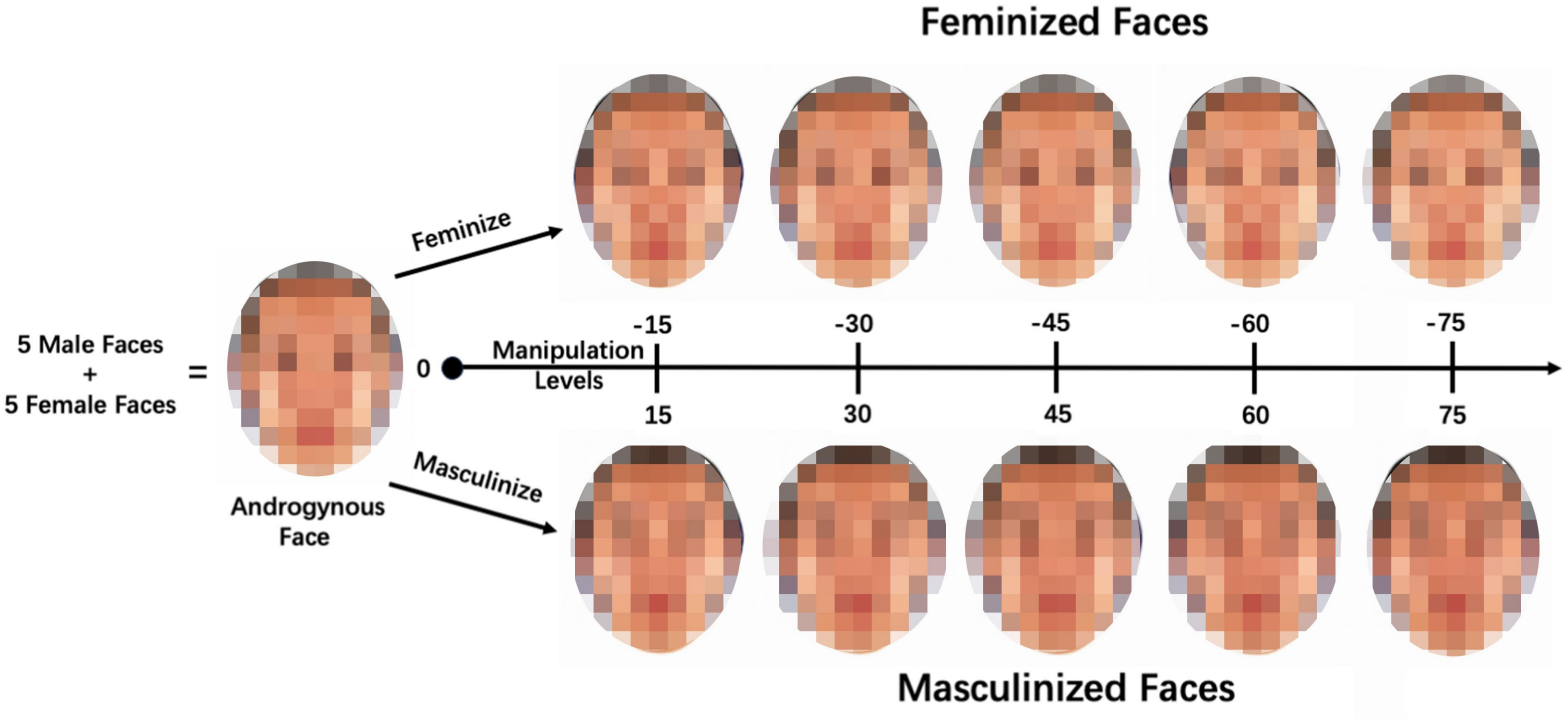
One series of the face stimuli. An androgynous face was synthesized from five male and five female faces. Next, based on the androgynous face, we generated five versions of a female face image (with standardized feminization levels of 15, 30, 45, 60, and 75%) and five versions of a male face image (with standardized masculinization levels of 15, 30, 45, 60, and 75%).

To objectively manipulate the sexual dimorphism of the face shape, we employed prototype-based image transformations (Tiddeman et al., 2001). The skin color and texture of the original face image were maintained. The selected five androgynous faces were subjected to alterations, where 15, 30, 45, 60, and 75% of the linear differences in the face shape between the symmetrized female and male prototypes were either added or subtracted. This iterative process resulted in the creation of 50 face images with standardized sex information. Specifically, for each androgynous face, there were five versions of a male face image (masculinized by 15, 30, 45, 60, and 75%) and five versions of a female face image (feminized by 15, 30, 45, 60, and 75%), as shown in Figure 1.

In conclusion, the five androgynous faces generated 50 face images with standardized sex information, plus five gender-neutral face images (i.e., the five androgynous faces), resulting in a total of 55 face images used in the current study.

### 2.3 Procedure

**Figure 2:**
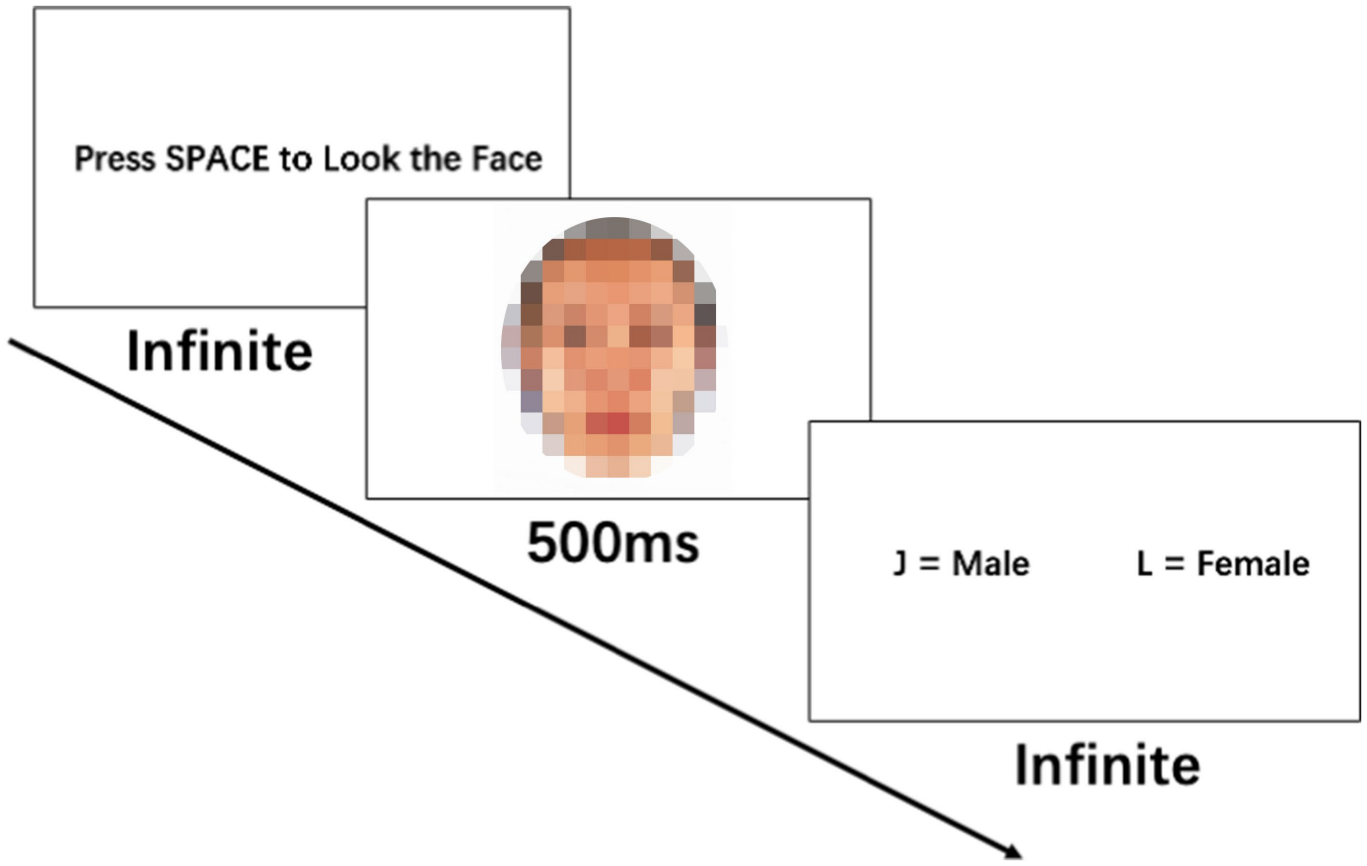
Task procedure.

Prior to participating in the study, all participants provided written informed consent. The gender judgment task was conducted on computers in quiet rooms. See Figure 2 for the task procedure. After preparing, the participants pressed the spacebar and were presented with one face at a time for a duration of 500 ms. They were then required to determine the sex of the displayed face. This process was repeated 55 times, with a total of 55 unique face image stimuli shown in a randomized order within each block. Each participant completed two identical blocks. The participants’ height (Mean = 173.87 cm, SD = 4.24 cm) and weight (Mean = 68.77 kg, SD = 13.38 kg) were measured before the experiment using a ruler and a weighing scale, and the BMI values were calculated (Mean = 22.68 kg/m^2^, SD = 3.96 kg/m^2^).

## 3 Results

The responses in the gender judgment task were analyzed using mixed binary logistic regression in R version 4.3.2 (R Core Team, 2023) with lmerTest version 3.1.0 (Kuznetsova et al., 2017). To avoid multicollinearity, we analyzed the height, weight, and BMI values separately. In each model, the response (dependent variable, DV) was the binary choice (dummy coding for the face gender judgment: 0 = female, 1 = male). The height, weight, and BMI values were standardized by transforming them into z-scores. The face manipulation levels were coded as follows: 0.75 = 75% increased masculinization, 0.60 = 60% increased masculinization, 0.45 = 45% increased masculinization, 0.30 = 30% increased masculinization, 0.15 = 15% increased masculinization, 0 = sex neutral (i.e., androgynous faces), −0.15 = 15% increased femininization (i.e., 15% decreased masculinization), −0.30 = 30% increased femininization, −0.45 = 45% increased femininization, −0.60 = 60% increased femininization, and − 0.75 = 75% increased femininization.

In the height model, the DV was the response and the independent variables (IVs) were height (z-scored), manipulation levels, and their interaction. Random intercepts were participant ID and base face ID. Random slopes were specified maximally, following Barr et al. (2013) and Barr (2013). This type of analysis takes into account variations in the effects of shape manipulations across stimuli items (in this study, each base face/androgynous face; Barr et al., 2013).

The weight model and the BMI model were identical to the height model, except that height was replaced with weight (z-scored) and BMI (z-scored), respectively.

### 3.1 Height

**Figure 3:**
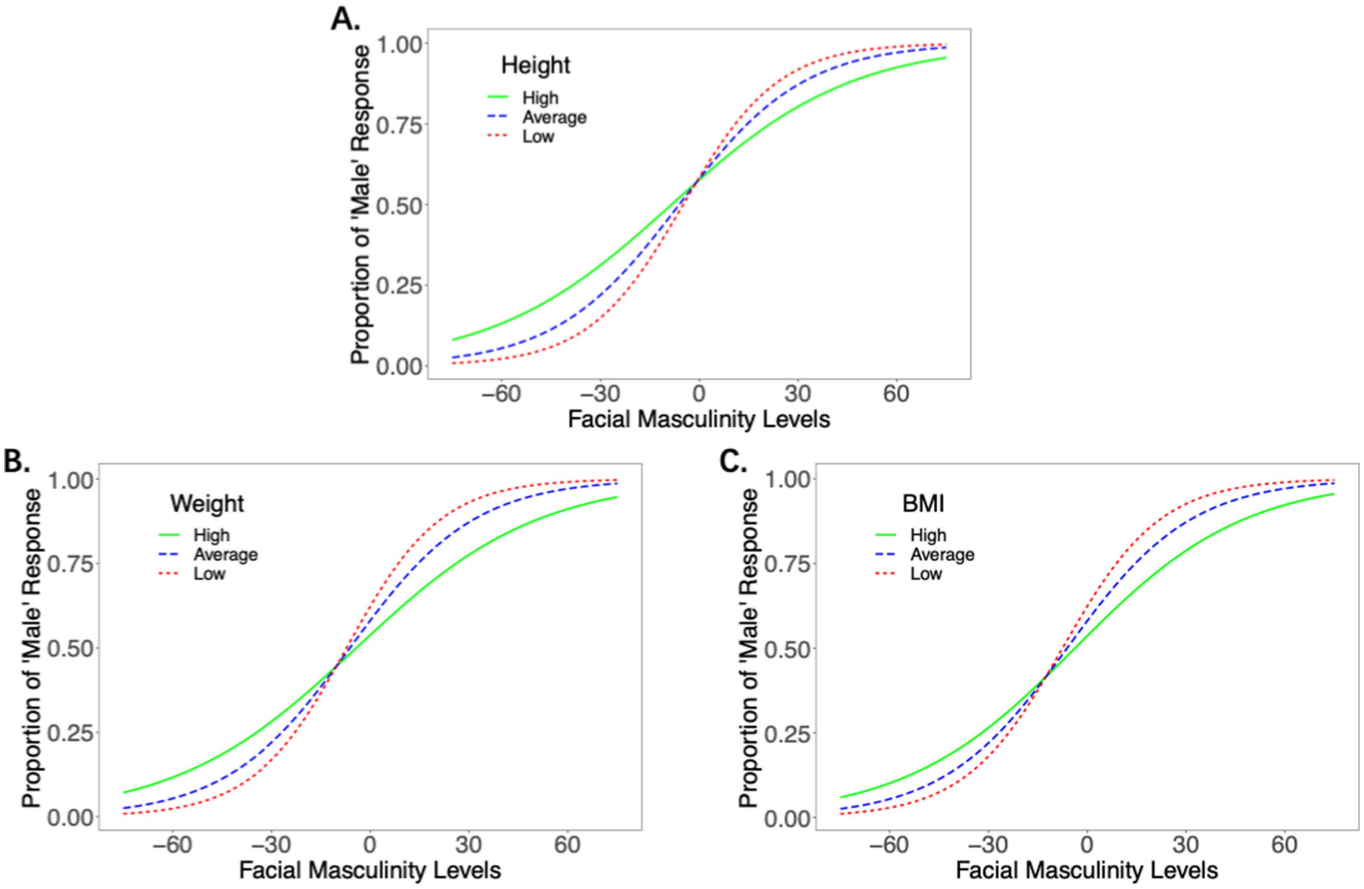
The effect of the individuals’ height **(A)**, weight **(B)**, and BMI **(C)** on their sensitivity to sexually dimorphic facial features in gender judgment. Low height/weight/BMI = 3 SD below the mean, high height/weight/BMI = 3 SD above the mean.

There was a main effect of the manipulation levels (*beta* = 3.98, *SE* = 0.13, *z* = 31.13, *p* < 0.001, *OR* = 53.77, [41.84, 69.11]), indicating that the manipulation significantly influenced the face gender judgment. The main effect of height (z-scored) was not significant (*beta* = −0.01, *SE* = 0.09, *z* = −0.06, *p* = 0.956, *OR* = 0.99, [0.83, 1.19]). The interaction between the manipulation level and the participants’ height was significant (*beta* = −0.41, *SE* = 0.14, *z* = −2.84, *p* = 0.004, *OR* = 0.66, [0.50, 0.88]), indicating that the participants’ height negatively influenced their sensitivity to sexually dimorphic facial features in gender judgment. Figure 3A shows that, compared to the individuals of higher stature, individuals of shorter stature exhibited higher sensitivity to sexually dimorphic facial features when judging others’ gender.

### 3.2 Weight

There was a main effect of the manipulation levels (*beta* = 3.98, *SE* = 0.13, *z* = 31.11, *p* < 0.001, *OR* = 53.80, [41.86, 69.16]), and the interaction between the manipulation level and the participants’ weight was significant (*beta* = −0.42, *SE* = 0.14, *z* = −2.99, *p* = 0.003, *OR* = 0.66, [0.50, 0.87]), suggesting that the participants’ weight negatively influenced their sensitivity to sexually dimorphic facial features in gender judgment. The main effect of weight (z-scored) was not significant (*beta* = −0.06, *SE* = 0.09, *z* = −0.63, *p* = 0.529, *OR* = 0.94, [0.79, 1.13]). Figure 3B shows that, compared to individuals with higher weight, individuals with lower weight exhibited higher sensitivity to sexually dimorphic facial features when judging others’ gender.

### 3.3 BMI

There was a main effect of the manipulation levels (*beta* = 3.98, *SE* = 0.13, *z* = 30.66, *p* < 0.001, *OR* = 53.73, [41.65, 69.32]), and the interaction between the manipulation level and the participants’ BMI was significant (*beta* = −0.36, *SE* = 0.14, *z* = −2.54, *p* = 0.011, *OR* = 0.70, [0.53, 0.92]), suggesting that the participants’ BMI negatively influenced their sensitivity to sexually dimorphic facial features in gender judgment. The main effect of BMI (z-scored) was not significant (*beta* = −0.06, *SE* = 0.09, *z* = −0.65, *p* = 0.517, *OR* = 0.94, [0.79, 1.13]). Figure 3C shows that, compared to individuals with higher BMI, individuals with lower BMI exhibited higher sensitivity to sexually dimorphic facial features when judging others’ gender.

### 3.4 Sexual dimorphism of the face group/face sex

For the new models, we set accuracy as the DV, which was dummy-coded as follows: 1 = correct (i.e., judgment aligned with the sexual dimorphism direction) and 0 = wrong (i.e., judgment did not align with the sexual dimorphism direction). Accuracy for androgynous faces was coded as 0, as they did not have a definitive corresponding answer. Indeed, coding androgynous faces as either 0 or 1 yielded the same result as the average response to the androgynous faces was close to the chance level (i.e., 0.5). Moreover, the face manipulation levels were recoded from 0 to 0.75 (representing from 0 to 75%) without considering sexual dimorphism direction. The sexual dimorphism of the face group was introduced as a new variable, effectively coded as follows: −0.5 = feminized group and 0.5 = masculinized group. The height, weight, and BMI values were entered as control variables in separate models. Random intercepts were participant ID and base face ID. Random slopes were specified maximally, following Barr et al. (2013) and Barr (2013).

**Figure 4:**
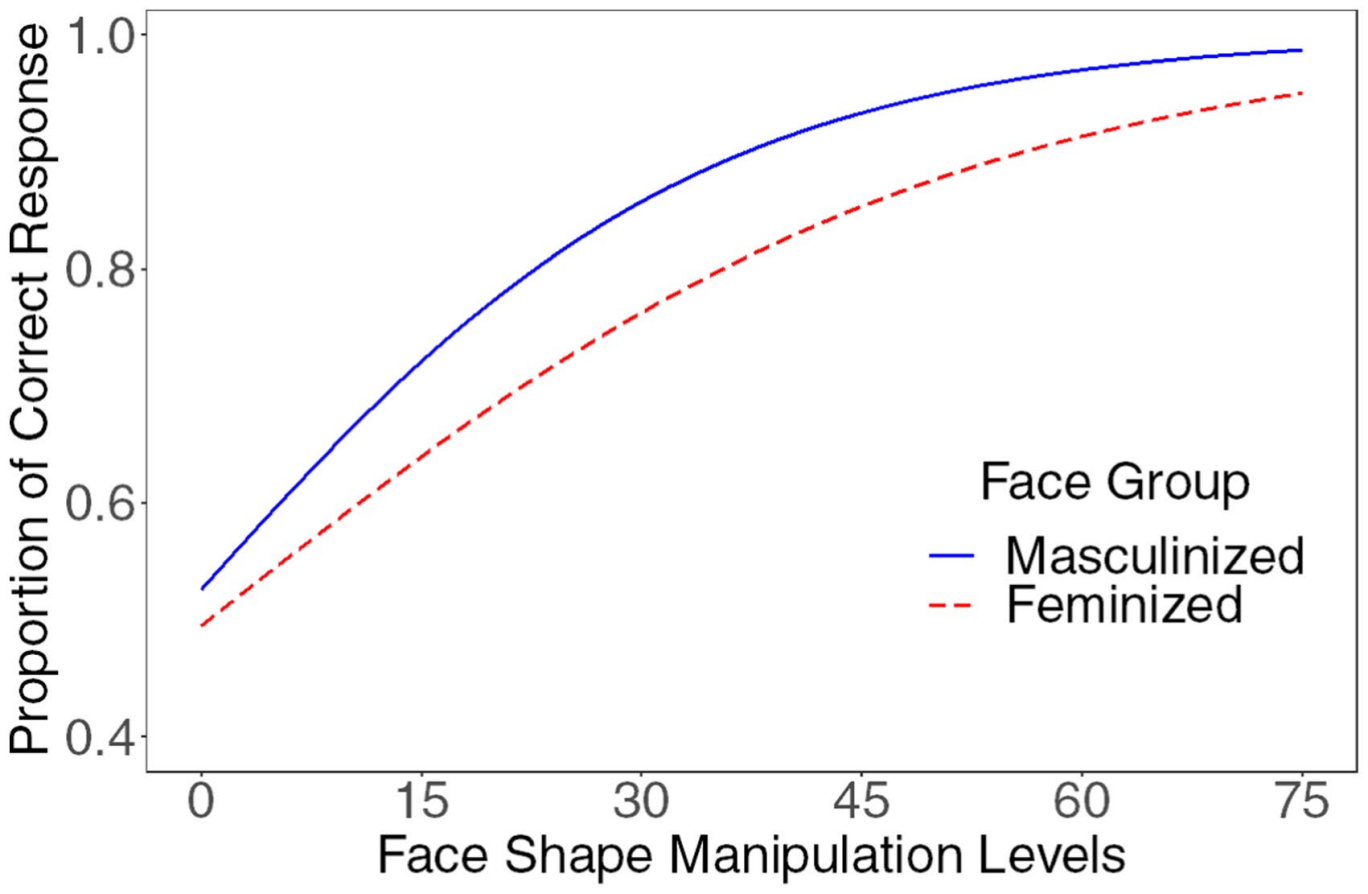
Compared to feminized faces, participants demonstrated greater sensitivity to sexually dimorphic facial features on masculinized faces.

In the following analyses, height was entered as a control variable. The main effect of the manipulation levels was significant (*beta* = 3.60, *SE* = 0.11, *z* = 33.60, *p* < 0.001, *OR* = 36.63, [29.69, 45.20]). The interaction between the manipulation level and the sexual dimorphism of the face group was significant (*beta* = 1.27, *SE* = 0.34, *z* = 3.71, *p* < 0.001, *OR* = 3.56, [1.82, 6.95]), suggesting that participants exhibited varying sensitivity to sexually dimorphic facial features between the masculine face group and the feminine face group. Specifically, compared to feminized faces, participants demonstrated greater sensitivity to sexually dimorphic facial features on masculinized faces (please see Figure 4). The interaction between the manipulation level and height was significant, *beta* = −0.45, *SE* = 0.10, *z* = −4.47, *p* < 0.001, *OR* = 0.64, [0.53, 0.78], replicating the previous results that the participants’ height negatively influenced their sensitivity to sexually dimorphic facial features in gender judgment. No other effects were significant (all absolute *betas* < 0.56, all absolute *z*s < 1.52, all *p*s > 0.129).

However, when the control variable of height was removed from the model, the interaction between the manipulation level and the sexual dimorphism of the face group was non-significant, *beta* = 0.64, *SE* = 1.15, *z* = 0.56, *p* = 0.579. This finding indicated that the influence of the face group on the sexually dimorphic cues was significant only when body size was controlled for.

In addition, the abovementioned analysis procedure was also applied to weight and BMI, and the results for weight and BMI showed a similar pattern to those for height. Please see the Supplementary material for details.

## 4 General discussion

The present study aimed to investigate individual differences in sensitivity to sexually dimorphic cues that signal gender, with a particular focus on the influence of men’s body size. The results showed that men’s height, weight, and BMI were negatively associated with their sensitivity to sexually dimorphic facial features in gender perception. This finding indicates that men with larger body sizes are less sensitive to sexually dimorphic facial features. Moreover, the results also showed that men were more sensitive to the sexually dimorphic cues on male faces than on female faces.

Previous research on dominance in men has revealed that men with large body sizes are less sensitive to sexually dimorphic cues. Studies have shown that taller and more dominant men are also less sensitive to sexually dimorphic cues when judging other men’s dominance (Watkins et al., 2010a,b). Facial masculinity, which is strongly associated with perceived and actual dominance, may play a role in this relationship (e.g., Fink et al., 2007; Von Rueden et al., 2008; Boothroyd et al., 2007; Jones et al., 2010).

Men with larger body sizes are generally more formidable and attractive (Sell et al., 2009; Kurzban and Weeden, 2005). More formidable and attractive men may face fewer consequences for incorrectly judging sexually dimorphic cues in other men and women since they may be better equipped to handle the potential costs of engaging in aggressive conflict with other men and may have more opportunities to attract women. This leads to a reduced need for accurate perception of sexually dimorphic cues. Given that larger men are less sensitive to sexually dimorphic cues, they may be less sensitive to social cues related to sexually dimorphic cues. There is evidence that more formidable men are more likely to underestimate other men’s formidability (Fessler and Holbrook, 2013; Fessler et al., 2014) and tend to exhibit lower levels of psychological defensiveness toward other men, as reflected in their higher ratings of other men’s attractiveness and trustworthiness (Macapagal et al., 2011) and lower levels of jealousy (Buunk et al., 2008).

Furthermore, the current study also found that men displayed greater sensitivity to the sexually dimorphic cues on male faces than on female faces. This finding suggests that men might encounter greater intrasexual rather than intersexual selection pressures and consequently exhibit greater sensitivity to sexually dimorphic cues on men’s faces. This finding aligns with that of another study, which found that men’s interpretations of sexually dimorphic cues mainly serve to reduce the costs of making mistakes during intrasexual, rather than intersexual, interactions (Watkins et al., 2010b).

There are limitations in the current study. First, the finding that men exhibited greater sensitivity to sexually dimorphic cues on male faces compared to female faces was observed only when controlling for men’s body size. This finding means that the results should be interpreted with caution. Second, the current study only focused on men, limiting the generalizability of the findings to women. Future research should investigate women’s gender perception.

In conclusion, the present study extends our knowledge of how individual differences in sensitivity to sexually dimorphic cues affect perceptions of gender. The findings suggest that men with larger bodies are less sensitive to sexually dimorphic facial cues in gender perception. The present study also found that men showed greater sensitivity to sexually dimorphic features on men’s faces compared to women’s faces. Future studies may explore the underlying psychological mechanisms driving these findings.

## Data Availability

The raw data supporting the conclusions of this article will be made available by the authors, without undue reservation.
