# Supplementary material for: Big bodies, blurred lines: the impact of male body size on sensitivity to sexually dimorphic facial features
Source: Front Psychol. 2025 Apr 17;16:1531324. doi: 10.3389/fpsyg.2025.1531324 (PMC12043585; doi:10.3389/fpsyg.2025.1531324)
Supplement: Supplementary file 1 [file Table_1.docx]

In the following analyses, weight was entered as a control variable. The main effect of manipulation levels was significant, *beta* = 3.59, *SE* = 0.11, *z* = 33.70, *p* < .001, *OR* = 36.23, [29.40, 44.64]. The interaction between manipulation level and sexual dimorphism of face group was significant, *beta* = 1.22, *SE* = 0.34, *z* = 3.57, *p* < .001, *OR* = 3.38, [1.73, 6.58], suggesting that participants have different sensitivity to sexually dimorphic facial features between masculine face group and feminine face group. Specifically, compared to feminized faces, participants demonstrated greater sensitivity to sexual dimorphic facial features on masculinized faces (please see Fig. S1). The interaction between manipulation level and weight was significant, *beta* = -0.39, *SE* = 0.09, *z* = -4.24, *p* < .001, *OR* = 0.68, [0.57, 0.81], which replicated results above that participant’s weight negative influence their sensitivity of sexual dimorphic facial feature in gender judgment. No other effects were significant (all absolute *betas* < 0.16, all absolute *z*s < 1.21 , all *p*s > .224).

Fig. S1 Weight entered as control variable

In the following analyses, BMI was entered as a control variable. The main effect of manipulation levels was significant, *beta* = 3.57, *SE* = 0.11, *z* = 33.71, *p* < .001, *OR* = 36.23, [29.40, 44.64]. The interaction between manipulation level and sexual dimorphism of face group was significant, *beta* = 1.19, *SE* = 0.34, *z* = 3.51, *p* < .001, *OR* = 3.38, [1.73, 6.58], suggesting that participants have different sensitivity to sexually dimorphic facial features between masculine face group and feminine face group. Specifically, compared to feminized faces, participants demonstrated greater sensitivity to sexual dimorphic facial features on masculinized faces (please see Fig. S2). The interaction between manipulation level and BMI was significant, *beta* = -0.32, *SE* = 0.09, *z* = -3.45, *p* < .001, *OR* = 0.68, [0.57, 0.81], which replicated results above that participant’s BMI negative influence their sensitivity of sexual dimorphic facial feature in gender judgment. No other effects were significant (all absolute *betas* < 0.15, all absolute *z*s < 1.27 , all *p*s > .205).

Fig. S1 BMI entered as control variable
